# Patient Reported Differences in Dry Eye Disease between Men and Women: Impact, Management, and Patient Satisfaction

**DOI:** 10.1371/journal.pone.0076121

**Published:** 2013-09-30

**Authors:** Debra A. Schaumberg, Miki Uchino, William G. Christen, Richard D. Semba, Julie E. Buring, Jim Z. Li

**Affiliations:** 1 Division of Preventive Medicine, Brigham & Women’s Hospital, Harvard Medical School, Boston, Massachusetts, United States of America; 2 Department of Ophthalmology & Visual Sciences, Center for Translational Medicine, John A. Moran Eye Center, University of Utah School of Medicine, Salt Lake City, Utah, United States of America; 3 Schepens Eye Research Institute, Department of Ophthalmology, Harvard Medical School, Boston, Massachusetts, United States of America; 4 Department of Epidemiology, Harvard School of Public Health, Boston, Massachusetts, United States of America; 5 Department of Ophthalmology, School of Medicine, Keio University, Tokyo, Japan; 6 The Wilmer Eye Institute, Johns Hopkins University School of Medicine, Baltimore, Maryland, United States of America; 7 Global Outcomes Research, Pfizer, Inc., San Diego, California, United States of America; Johns Hopkins Bloomberg School of Public Health, United States of America

## Abstract

**Purpose:**

Dry eye disease affects women twice as often as men, but there is little information on whether dry eye treatments, treatment satisfaction, or the impact of dry eye disease on patients’ lives and vision might differ by sex.

**Design:**

Questionnaire survey of 4000 participants in the Women’s Health Study and the Physicians’ Health Studies I and II with a prior report of a diagnosis of DED.

**Methods:**

Among participants who re-confirmed a diagnosis of dry eye disease, we assessed symptoms, treatments, patient satisfaction and impact of dry eye disease, and analyzed differences between men and women using regression models.

**Results:**

The final study population consisted of 1,518 women (mean age 70.7 years) and 581 men (mean age 76.7 years), with a mean reported duration of dry eye disease of 10.5 years and 10.1 years, respectively. The frequency and severity of dry eye disease symptoms were higher among women (each P<0.0001), and women reported a greater impact on everyday activities (P<0.0001). Women were more likely to use artificial tears (P<0.0001) use them more often (P<0.0001), and to use Restasis® (P<0.0001), omega-3 fatty acids (P=0.0006), and have punctal occlusion (P=0.005). Women spent more money per month on dry eye treatments (P<0.0001), but reported greater dissatisfaction with treatment side effects (P=0.001), and the amount of time before treatments started working (P=0.03).

**Conclusions:**

These data show that dry eye disease is generally experienced as being more severe among women, having a greater impact on their self-assessed well-being.

## Introduction

Dry eye disease (DED) is an important public health problem characterized by a deficiency in the quantity and/or quality of tears, an unstable tear film, ocular surface damage, and bothersome symptoms such as ocular irritation, dryness, fatigue, and fluctuating visual disturbances. It results in increased risk of ocular surface infections and the bothersome symptoms frequently interfere with activities such as reading, working on a computer, and driving a car [1-4]. Studies over the past decade have identified older age, female sex, reduced androgen levels, exogenous estrogen use, imbalances in the dietary intake of essential fatty acids, certain antihypertensive medications, and antidepressant medications as risk factors for dry eye disease (DED) [2,5-7]. Large-scale studies of participants’ in the Women’s Health Study (WHS) and Physicians’ Health Studies (PHS) estimated that 3.25 million US women, and 1.68 million men aged 50 and older are affected with DED [7,8]. The prevalence of DED is higher in several Asian populations, ranging from 21% to 52.4% [9-14]. Although DED is now known to be more common in women [2,7,8], there is little information on whether the severity of DED, its impact on patients’ lives and vision, and how patients are treated may be different for women versus men. This study was undertaken to provide information on such factors.

## Design and Methods

### Study Subjects

Subjects for this study came from two large national longitudinal studies of healthcare professionals in the US: the Women’s Health Study (WHS) [15] and the Physicians’ Health Studies (PHS) I and II [16,17].

The WHS was a randomized, double-blind, placebo-controlled trial conducted among 39,876 US female health care professionals that was undertaken to assess the benefits and risks of low-dose aspirin and vitamin E in the primary prevention of cardiovascular disease and cancer. The original PHS (PHS I) was a randomized, double-blind, placebo-controlled trial to test the effects of low-dose aspirin and beta-carotene in the primary prevention of cardiovascular disease and cancer among 22,071 US male physicians. The second PHS (PHS II) is a randomized, double-blind, placebo-controlled factorial trial of alternate day beta-carotene, alternate day vitamin E, daily vitamin C, and a daily multivitamin in the prevention of cardiovascular disease, total and prostate cancer, and age-related eye diseases (cataract and macular degeneration) among 14,641 US male physicians aged 55 years and older, including 7,641 participants from the original PHS and 7,000 newly recruited male physicians. The remaining PHS I participants who elected not to enroll in PHS II continue to be followed by annual questionnaires. The WHS, PHS I, and PHS II are conducted by mail. The active treatment phase has ended for both the WHS and PHS I, but observational follow-up of these cohorts is ongoing. Because of their training in medicine or other areas of healthcare, participants have reliably reported specific details about their health. Participants have received questionnaires every year on which they reported their compliance with study regimens and any side effects, as well as recorded a number of health-related exposures and any health outcomes experienced over the previous year. We previously assessed diagnoses of DED and DED symptoms using a short questionnaire among participants of WHS (aged ≥49) and PHS I and II (aged ≥55) [7,8,18]. Of the participants who reported a diagnosis of DED or severe dry eye symptoms in the above studies [4-8], 4000 participants were selected and sent an expanded questionnaire to assess DED symptoms, co-morbid conditions, treatments, risk factors, and disease impact. We selected participants who had previously reported a diagnosis of DED starting with the cases with the longest duration of disease in order to achieve a study population of approximately 2,500 female participants and 1,500 male participants. This report includes only the subset of participants who confirmed in the present study their prior report of having a diagnosis of DED. The method for identifying cases of DED was previously validated and shown to accurately identify cases of DED diagnosed by standard clinical tests [17].

The WHS, PHS I and II, and dry eye ancillary study was approved by the Human Subjects Committee at Brigham and Women’s Hospital, and all subjects gave written informed consent.

### Measurements

#### Characteristics of the study participants

The authors collected data on the demographic characteristics of study participants from available questionnaire data in each cohort. Data included participant age, sex, self-reported race/ethnicity, professional qualification, and highest education level. Information on the date of diagnosis of dry eye was also obtained from study participants, and was used to calculate the duration of DED as of January 2011.

#### DED symptom severity, impact on daily activities

We administered a supplementary questionnaire to all participants selected for participation in this study. The questionnaire included a number of questions concerning symptoms associated with DED, including 1) The Ocular Surface Disease Index (OSDI, ©1995 Allergan, Inc) [19], and 2) The Symptom Assessment in Dry Eye (SANDE) [20]. The OSDI consists of 12 questions to assess ocular symptoms (the Ocular Symptoms subscale), their impact on patient vision-related functioning (Vision Related Function subscale), and environmental factors triggering the symptoms (Environmental Triggers subscale). The three subscales and an overall OSDI Total score were scored according to the user manual, each with a 100-point scale ranging from ‘None’ to ‘Most severe’. Based on the recommended cutoffs for OSDI Total score, we grouped the subjects into mild, moderate or severe (OSDI Total score = 13-22, 23-32, or 33 and higher). The SANDE is a simple dry eye instrument containing two items measuring the frequency and severity of symptoms, each was assessed on a 100 mm visual analog scale (VAS) ranging from ‘Rarely/Very mild’ to ‘All the time/Very severe’ and scored from 0 to 100, from which a SANDE Total score (also ranging from 0 to 100) was calculated as the square-root of the product of the two item scores [20]. Based on the SANDE total score, we grouped the subjects into three categories: Mild (<40), Moderate (40-50), or Severe (>50).

In addition to the OSDI and SANDE, we also included a question on fluctuating/unstable vision, ability to perform the participant’s work/profession, as well as a series of questions regarding feelings of calm/peacefulness, energy level, feeling downhearted and depressed, and interference with social activities. Participants were also asked to assess the overall degree to which eye symptoms limited daily activities by using a 10-point scale ranging from ‘Not at all’ to ‘Severely’.

#### DED treatments and satisfaction with DED treatment

A comprehensive list of DED treatments was provided and participants were asked to check selected types of dry eye treatments they used. Level 2 or higher treatments were defined according to the recent report of the Dry Eye Workshop (DEWS) [3]. We also asked about the amount of money participants spent each month on dry eye treatments.

We measured satisfaction with DED treatment using a series of 8 questions on the ability of treatments to relieve symptoms, time it takes for treatments to start working, length of symptom relief, ease of use, convenience, side-effects, and overall treatment satisfaction. Responses were recorded on a 5-point scale ranging from ‘Extremely satisfied’ to ‘Extremely dissatisfied’.

### Statistical Analysis

We compared data from the dry eye questionnaire between respondents from the WHS (all women), and the PHS I and PHS II (all men). To test for differences between men and women, we used t-tests for continuous variables (current age, duration of DED, OSDI and SANDE scores), Chi-square tests for dichotomous variables, and Cochran-Armitage Trend tests for ordinal variables. To adjust for age differences between the cohorts, we used least square regression models for continuous variables, and constructed multivariable logistic regression models for each dichotomous factor of interest, and tested whether sex was associated with that factor after controlling for current age as a continuous variable. For factors with >2 levels of ordered responses (ordinal variables), we fit proportional odds models to test for associations with sex, while simultaneously adjusting for possible associations with age. For these models, we initially fit models using all original response levels and tested the proportional odds assumption using the score test. If the proportional odds assumption was violated, we fit a new model based on collapsing response levels from 5 or more to 3 and re-tested the proportional odds assumption. From each model, we obtained odds ratios (OR) and their corresponding ninety-five percent confidence intervals (CI) for the association of age and sex with each factor.

## Results

### Demographic and Clinical Characteristics of the Respondents

Of the 4000 WHS and PHS participants to whom we mailed the survey questionnaire, 3390 returned the questionnaire (85% response rate). Of the 3390 respondents, 2099 (62%) had previously reported a clinical diagnosis of dry eye and confirmed a diagnosis of dry eye on the current questionnaire. This constituted the dry eye sample for this paper.

Of the 2099 respondents who confirmed a diagnosis of dry eye, 581 (28%) were men and 1518 (72%) were women. About 91% men and 96% women were white. By design of the source studies, all men were physicians with MD or equivalent degrees, whereas the women were all health professionals, including three quarters who were registered nurses. Women were 6 years younger on average (mean age at diagnosis=60 y) compared to men (mean age at diagnosis=66 y) at the time they were first diagnosed with DED, but had similar duration of DED at the time of the survey (Table 1). There were no differences between women and men in self-reported general health (p=0.74) and general eye health (p=0.48).

**Table 1:** Characteristics of the study participants.

	Men	Women	P value
Sample size (N)	581	1518	
Race (%)			<0.0001
White	91.1	96.0	
Hispanic	1.9	1.4	
African American/Black	0.9	1.2	
Asian	4.7	1.0	
Other	1.5	0.4	
Professional qualification (%)			<0.0001
MD or equivalent	100	2.2	
RN	0	77.1	
LPN/LVN	0	13.0	
Other	0	7.6	
Highest educational level			<0.0001
Licensed/Registered nurse training	0	55.5	
BS nursing	0	24.7	
Master’s degree	0	14.9	
MD/doctoral degree	100	4.9	
Current Age (years; mean & SD)	76.7 (8.6)	70.7 (6.5)	<0.0001
Age at Diagnosis of DED (years; mean & SD)	66.1 (12.6)	60.0 (11.0)	<0.0001
Duration of dry eye (years; mean & SD)	10.1 (9.2)	10.5 (8.9)	0.42

### DED Symptom Severity, Impact on Daily Activities

**Figure 1:**
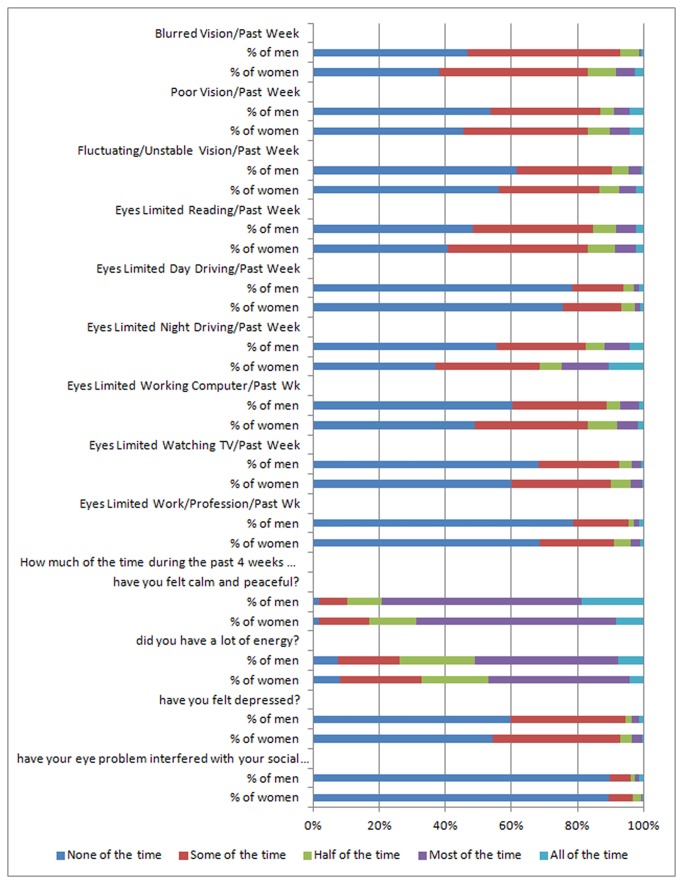
Impact of DED on quality of life measures, by sex. The chart depicts the percentage of women (N=1518) and men (N=581) with diagnosed dry eye for each category of response for questions related to quality of life.

As measured by OSDI subscale and overall scores (each P<0.0001), or by SANDE item and overall scores (each P<0.0001), women reported significantly higher levels of dry eye symptom severity (Table 2). When categorized by the OSDI overall score, 33.6% of women and 15.6% of men reported severe dry eye symptoms, with mean scores of 26.8 among women versus 18.2 among men. Using the SANDE overall score, dry eye symptoms of 39.1% of women and 17.9% of men were classified as severe and women reported a mean score of 42.3 compared with 27.2 among men. Overall, women also reported a significantly greater impact and worse quality of life from their disease (Table 3 and Figure 1). For example, women with DED reported significantly greater problems with vision (blurred vision, poor vision, and fluctuating/unstable vision), reading, driving at night, watching television, and working on a computer compared to men with DED. Women with DED were less likely than men with DED to report feeling calm and peaceful or having a lot of energy, and more likely to report feeling depressed.

**Table 2:** Current level of DED symptoms reported by men and women with diagnosed DED.

	Women	Men	P value
	Mean (SD)	Mean (SD)	
DED symptoms based on OSDI*†			
Ocular Symptoms subscale	29.0 (21.6)	18.5 (18.3)	<0.0001
Vision Related Function subscale	22.0 (19.9)	17.3 (19.3)	<0.0001
Environmental Triggers subscale	35.4 (29.3)	21.2 (23.7)	<0.0001
Overall score of OSDI	26.8 (18.7)	18.2 (16.4)	<0.0001
DED symptoms based on SANDE* ‡			
Frequency	48.4 (28.5)	32.4 (28.1)	<0.0001
Severity	39.4 (27.4)	24.9 (23.0)	<0.0001
Overall Score	42.3 (26.8)	27.2 (23.7)	<0.0001
How much dry eye symptoms limited daily activities§	3.2 (2.2)	2.6 (1.8)	<0.0001

* P-values are from linear regression models adjusted for age. Age was significantly inversely associated only with the OSDI Environmental Triggers subscale (P=0.0002)

† OSDI=The Ocular Surface Disease Index, a validated questionnaire to measure symptoms of DED. Scores range from 0 (no symptoms) to 100 (maximum symptoms).

‡ SANDE=The Symptom Assessment in Dry Eye questionnaire, a validated visual analogue scale to assess ocular surface discomfort in DED. Scores range from 0 to 100 with higher scores indicating worse symptoms.

§ A single question from our questionnaire; scores ranged from 0=Not at all - 10=Severely. This P-value is from a proportional odds logistic regression model adjusted for age.

**Table 3:** Impact of DED on quality of life measures among women versus men with diagnosed DED.

	OR (95% CI)*	P value*
How much dry eye symptoms limited daily activities§	1.50 (1.24-1.80)	<0.0001
Experienced the following during the past 4 weeks †		
Blurred vision	1.31 (1.07-1.60)	0.01
Poor vision	1.40 (1.14-1.72)	0.002
Fluctuating/unstable vision	1.27 (1.02-1.58)	0.03
Eye problem limited the following during the past 4 weeks †		
Reading	1.34 (1.10-1.64)	0.004
Driving during the day	1.27 (0.98-1.65)	0.08
Driving at night	2.76 (2.23-3.42)	<0.0001
Working with computer or bank machine (ATM)	1.65 (1.32-2.06)	<0.0001
Watching T.V.	1.50 (1.20-1.88)	0.0005
Your work/profession	1.35 (0.97-1.83)	0.05
How much of the time during the past 4 weeks †		
have you felt calm and peaceful?	0.62 (0.45-0.85)	0.003
did you have a lot of energy?	0.63 (0.50-0.79)	<0.0001
have you felt depressed?	1.28 (1.04-1.57)	0.02
have your eye problem interfered with your social activities?	1.22 (0.86-1.73)	0.26

* The odds ratio (OR) indicates the ratio of women’s odds over men’s, each estimated from a separate proportional odds logistic regression model adjusted for age. None of the models violated the proportional odds assumption.

§ Scores ranged from 0 (=Not at all) to 10 (=Severely).

† Response levels collapsed to 3 categories (None of the time, Some of the time, or Half/Most/All of the time)

### DED Treatments and Satisfaction with DED Treatment

**Figure 2:**
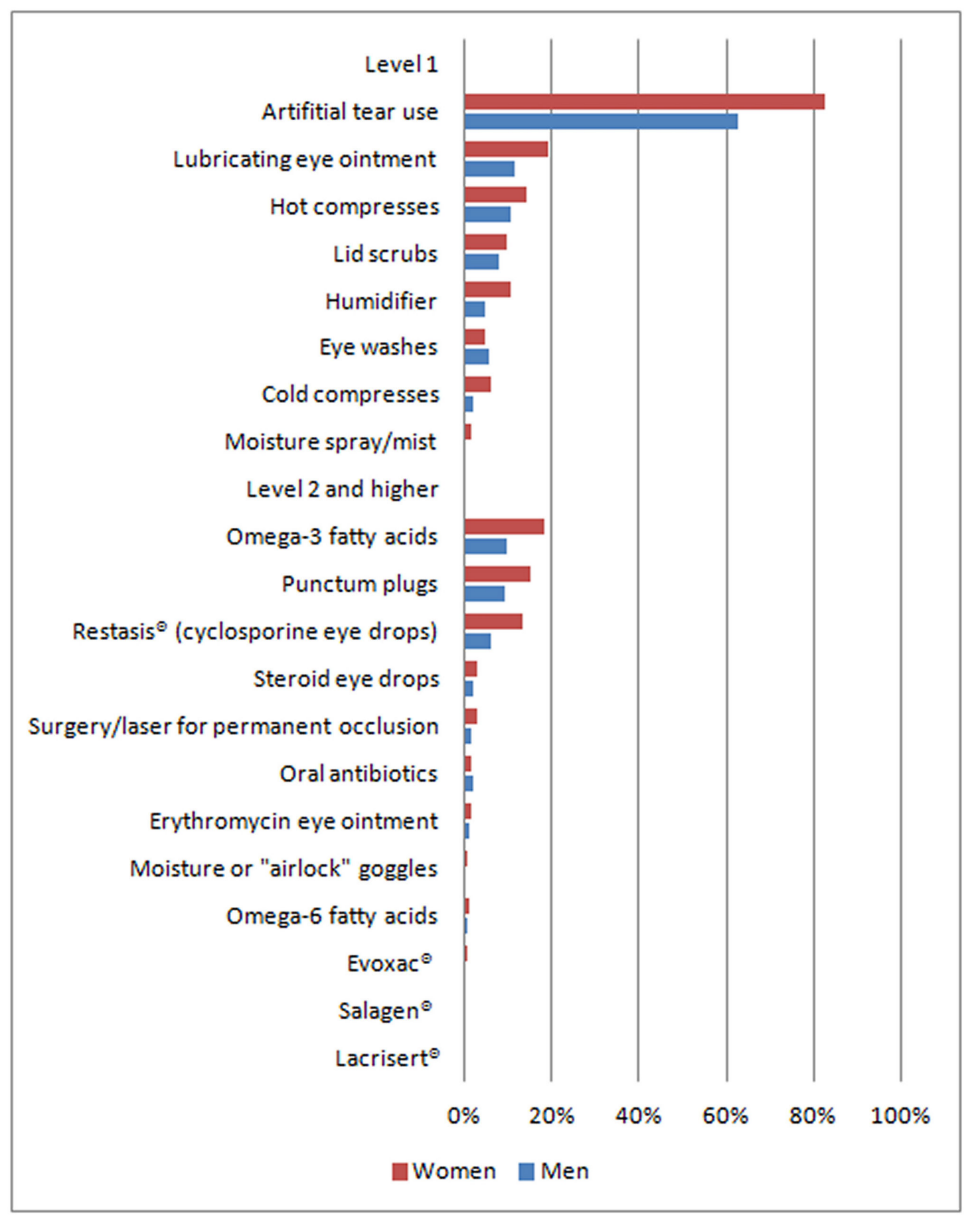
Reported use of DED treatments, by sex. The chart depicts the percentage of subjects reporting use of a number of available dry eye treatments, separately for women (N=1518) and men (N=581) with diagnosed dry eye.

Of the DED treatments we inquired about, the top three Level 1 treatments used were artificial tears (used by 82.8% women and 62.6% men), lubricating eye ointments (used by 19.2% women and 11.7% men), and hot compresses (used by 14.3% women and 10.7% men). The top three Level 2 and higher levels treatments were oral omega-3 fatty acids supplements (used by 18.6% women and 9.6% men), punctum plugs (used by 15.0% women and 9.1% men) and cyclosporine eye drops (Restasis®, used by 13.4% women and 6.4% men) (Figure 2). When comparing between women and men, and adjusting for age, we found significantly more women were using each of these 6 treatments (Table 4). Consistent with this, we also observed that women spent more money, on average, per month on dry eye treatments (P<0.0001).

**Table 4:** Odds of use of DED treatments among women versus men with DED.

	OR (95% CI)*	P value*
***Level 1***		
Artificial tear use	3.46 (2.71-4.41)	<0.0001
Lubricating eye ointment	1.80 (1.33-2.43)	0.0001
Hot compresses	1.48 (1.07-2.04)	0.02
Lid scrubs	1.30 (0.90-1.88)	0.16
Humidifier	2.47 (1.59-3.83)	<0.0001
Eye washes	1.01 (0.64-1.59)	0.96
Cold compresses	2.91 (1.58-5.37)	0.0006
Moisture spray/mist	8.11 (1.07-61.24)	0.04
***Level 2 and higher***		
Omega-3 fatty acids	1.74 (1.27-2.39)	0.0006
Punctum plugs	1.55 (1.11-2.16)	0.01
Restasis® (cyclosporine eye drops)	2.17 (1.48-3.17)	<0.0001
Steroid eye drops	1.35 (0.68-2.67)	0.39
Surgery/laser for permanent punctal occlusion	1.57 (1.15-2.14)	0.005
Oral antibiotics	0.71 (0.34-1.49)	0.36
Erythromycin eye ointment	1.39 (0.60-3.22)	0.45
Moisture or "airlock" goggles	2.23 (0.46-10.68)	0.32

* The odds ratios (OR) indicate the ratio of women’s odds over men’s, each estimated from a separate logistic regression model adjusted for age.

**Figure 3:**
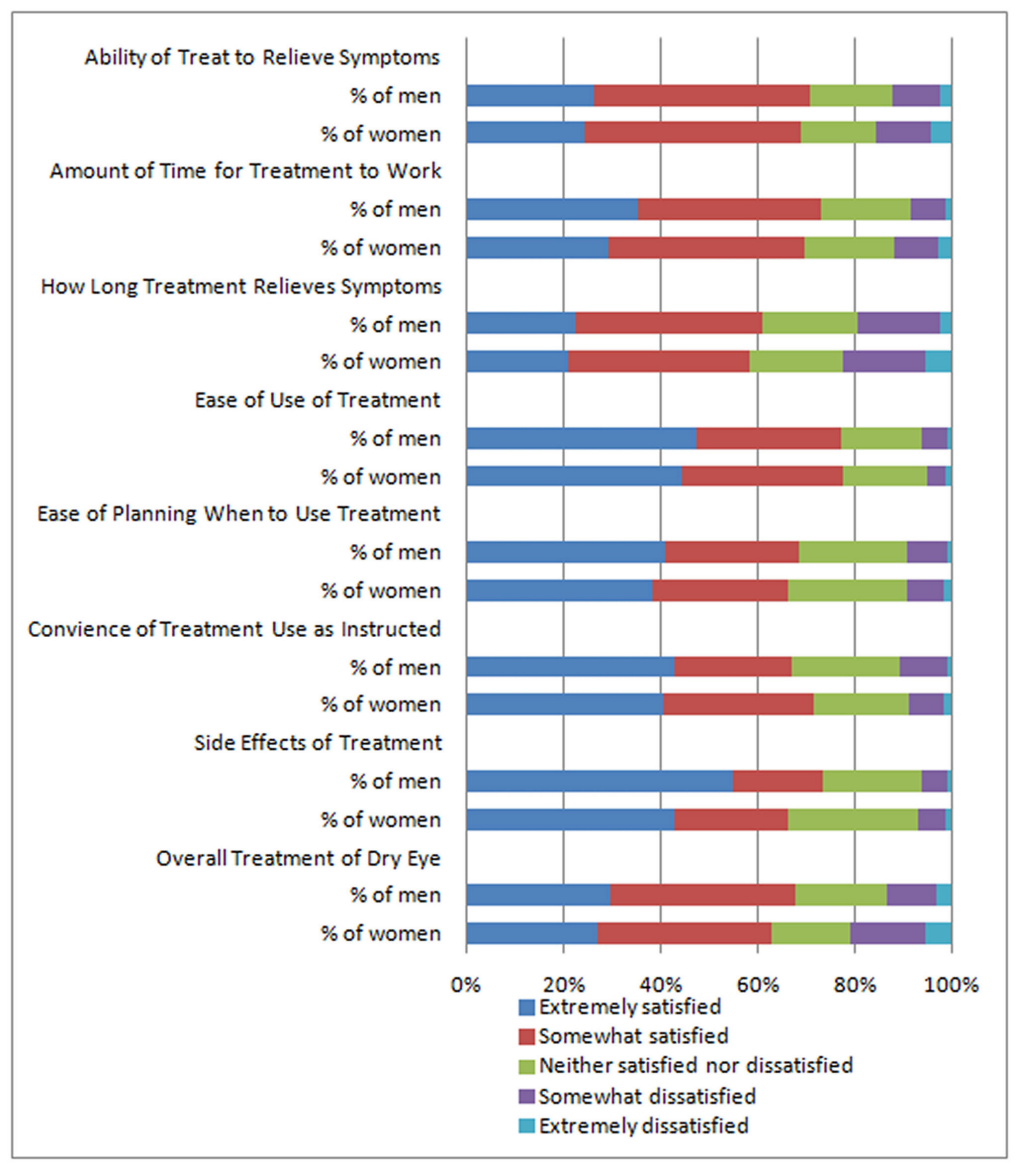
Satisfaction with DED treatments, by sex. The chart depicts participant satisfaction with using dry eye treatments, separately for women (N=1518) and men (N=581) with diagnosed dry eye.

In analyses of patient satisfaction with DED treatments, respondents in general were satisfied with the treatments’ ability to relieve symptoms, the amount of time for the treatments to work, and their ease of use and convenience, but less satisfied with the treatments’ duration of effect (how long treatments relieved symptoms) (Figure 3). When comparing women with men and adjusting for age in proportional odds models across the 5 categories of response (ranging from extremely dissatisfied to extremely satisfied), women expressed about the same level of general satisfaction with treatments, except for reporting greater dissatisfaction with treatment side effects, and the time it takes for the treatment to start working (Table 5). The proportional odds assumption was violated in 3 of these models, for the ability of the treatment to relieve symptoms, how long the treatment relieves symptoms, and the convenience of treatment use as instructed. In alternative models in which we collapsed responses into 3 categories of somewhat or extremely dissatisfied, neither satisfied nor dissatisfied, and somewhat or extremely satisfied, the proportional odds assumption was not violated for the model for convenience of treatment use as instructed, for which the model showed that women were significantly less likely to be dissatisfied with this aspect of their dry eye treatment (OR=0.74, CI=0.58-0.95).

**Table 5:** Comparison of Satisfaction with DED Treatments among Women versus Men.

	OR over 5 Categories (95% CI)*	P value*
Ability of Treatment to Relieve Symptoms†	1.10 (0.89-1.35)	0.39
Amount of Time for Treatment to Work	1.27 (1.03-1.57)	**0.03**
How Long Treatment Relieves Symptoms†	1.03 (0.83-1.27)	0.79
Ease of Use of Treatment	1.02 (0.82-1.26)	0.89
Ease of Planning When to Use Treatment	1.01 (0.82-1.24)	0.95
Convenience of Treatment Use as Instructed†	0.87 (0.70-1.07)	0.19
Side Effects of Treatment	1.43 (1.15-1.78)	**0.001**
Overall Treatment of Dry Eye	1.14 (0.93-1.40)	0.22

* The odds ratios (OR) indicate the ratio of women’s odds over men’s, each estimated from a separate proportional odds logistic regression model adjusted for age.

† The proportional odds assumption was violated in the models indicated.

## Discussion

Previous analyses from these cohorts demonstrated a significantly higher prevalence of DED among women compared to men in the US [7,8]. In the present study, we extend those observations to show that women are not only affected more often, but are also diagnosed at an average of 6 years younger age. Recent estimates from the United Nations Department of Economic and Social Affairs, showed that the average life expectancy in the US for women is 5 years longer than that of men. Therefore, given its chronic nature, an earlier diagnosis of DED might result in a longer duration of suffering with this disease for women. Our data also show that women’s self-reported experience with DED tends to be more severe, as they report higher levels of dry eye symptoms, greater use of dry eye treatments, and a greater adverse impact of DED on everyday activities including reading, driving, watching TV and working with a computer. Although there were no significant differences in reported overall levels of satisfaction with regard to dry eye treatments after controlling for age-related differences (younger individuals reported less satisfaction), women were more dissatisfied with treatment side effects, and the amount of time before treatments started working.

The study population of women and men with diagnosed dry eye included participants with few symptoms to those with severe dry eye symptoms. The percentage of women who could be categorized as having severe symptoms was about double that of men. Consistent with this, mean total OSDI and SANDE scores as well as all OSDI subscale scores were all higher among women. The cornea and ocular surface are richly innervated with sensory neurons that respond to a variety of stimuli, causing sensations of ocular surface irritation as well as stimulation of mechanisms designed to protect the ocular surface system. In dry eye, the sensory nerves may be damaged due to chronic inflammation or other mechanisms, giving rise to dry eye symptoms and also possibly contributing to disease chronicity. Thus, dry eye can be considered a type of chronic pain condition with much of its public health impact due to its symptoms. In that light, data from the present study are consistent with the general observation that, compared to men, women tend to report more intense, frequent, and longer duration pain [21]. It has been widely accepted that hormonal alterations such as administration and withdrawal of estrogens can increase risk for pain [22]. Recent clinical and epidemiologic findings also indicate that women are at increased risk for many choronic pain conditions, and women tend to report higher levels of acute procedural pain [22]. Prior meta-analyses also showed that there are sex differences in experimental pain sensitivity [23]. It is thought that such sex-related differences in pain are likely due to the interaction of biological differences between men and women (e.g. genetic or hormonal factors) with psychological and socio-cultural factors [22]. Future studies might focus on determining the underlying causes of the greater severity DED symptoms in women.

Previous studies have identified a number of functional consequences of dry eye including difficulties with tasks requiring sustained visual attention such as reading, driving at night, and using a computer [4]. Results from the present study indicate that women with DED report experiencing such limitations more frequently than men. Specifically, women reported a higher frequency of experiencing limitations of daily activities overall and specifically more frequent problems with reading, driving at night, working with a computer or bank machine, and watching TV compared to men. Women also reported more frequent blurred vision, poor vision, and fluctuating or unstable vision. In addition to these vision-related impacts, women with DED were less likely to feel calm and peaceful, less likely to have a lot of energy, and more likely to report anxiety, and to feel depressed.

A limitation of the study relates to our assessment of DED using self-reports of clinically diagnosed dry eye and self-reported information on its impact. However, we previously validated our questionnaire-based method of DED assessment and found a good balance of sensitivity and specificity versus commonly used clinical tests for DED [18], and we limited this analysis to respondents who confirmed their original report of DED on the most recent questionnaire. Although it remains possible that some of the men and women in our study may not have DED, use of the same methodology in both cohorts should have minimized any bias in comparisons between men and women. There were also differences in the degree of education and medical knowledge between the men and women in this study, and it is possible that differences in the degree of medical knowledge might have impacted reporting of some items. Finally, the underlying cohorts from which the study population was derived are not a random sample of the US population, and exclusion criteria for these two large-scale randomized prevention trial cohorts may have impacted the prevalence of some factors. Comparisons between men and women should remain valid, however, as the selection criteria were virtually identical for the two randomized trials.

The study benefits from a large sample size and in depth information including assessments of dry eye symptoms, co-morbid conditions, medication use, dry eye treatments, and measures of treatment satisfaction. There were no significant differences in the duration of DED between the men and women, which should minimize any differences that might arise with longer duration of disease. We controlled for age in order to control for possible confounding that might have arisen due to the older age of the PHS cohort.

DED has received recognition as a common ocular problem affecting older women more often than men, but there have been fewer data to describe possible differences in disease severity, impact, treatments, and treatment satisfaction between the sexes. The present study fills some of these gaps by characterizing a large group of men and women with DED. The study demonstrates that women with DED are diagnosed at a younger age, and report a higher level of symptoms, greater use of treatments, and lower satisfaction with some aspects of treatment compared to men. Women with DED also reported a greater impact of DED on everyday activities. Such findings are indicative of a greater impact of DED on quality of life among women. These findings also indicate the impact of DED extends beyond the eye and vision, and we hope might encourage clinicians to seek means of managing the “whole patient” so that the impact of DED can be minimized.
